# Dermoscopy of Amelanotic Melanoma in a Patient With Oculocutaneous Albinism

**DOI:** 10.5826/dpc.1003a51

**Published:** 2020-06-29

**Authors:** Belkis Uyar, Ömer Faruk Elmas, Asuman Kilitçi, Murat Tad

**Affiliations:** 1 Department of Dermatology and Venereology, Ahi Evran University, Kirşehir, Turkey; 2 Department of Pathology, Ahi Evran University, Kirşehir, Turkey

**Keywords:** oculocutaneous albinism, melanoma, dermoscopy

## Introduction

Oculocutaneous albinism (OCA) is a group of autosomal recessive disorders characterized by defective melanin biosynthesis due to full or partial reduction in tyrosinase activity, which results in congenital depigmentation or hypopigmentation of the hair, skin, and eyes despite the normal number of melanocytes. In OCA, reduced or absent protection of melanin leads to sensitivity to ultraviolet radiation and a predisposition to skin cancers. Dermoscopic features of melanoma in patients with OCA have been reported in a few case studies. Here we report dermoscopic findings of an invasive melanoma arising from nevus in a patient with OCA.

## Case Presentation

**Figure 1:**
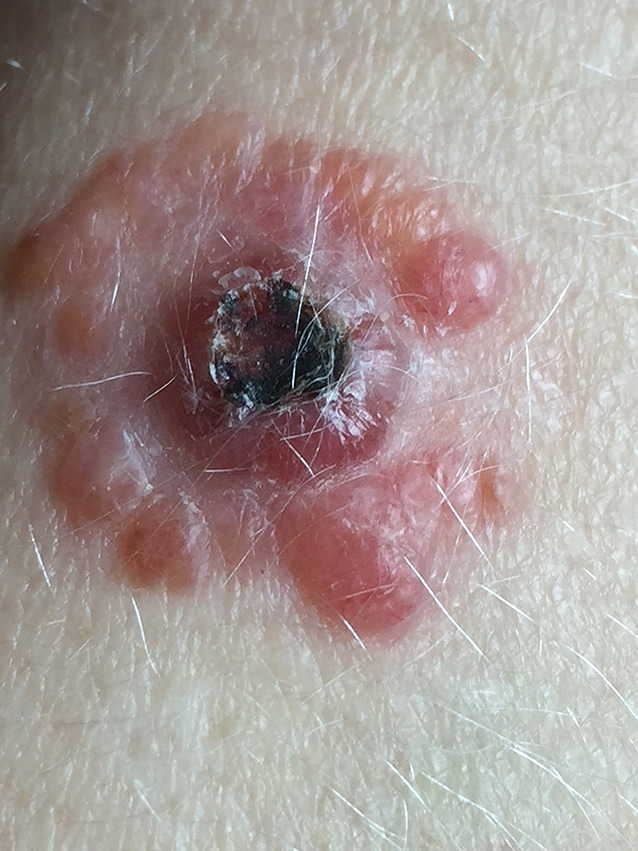
Multiple pinkish papules surrounding a main central tumoral lesion over the right forearm.

**Figure 2:**
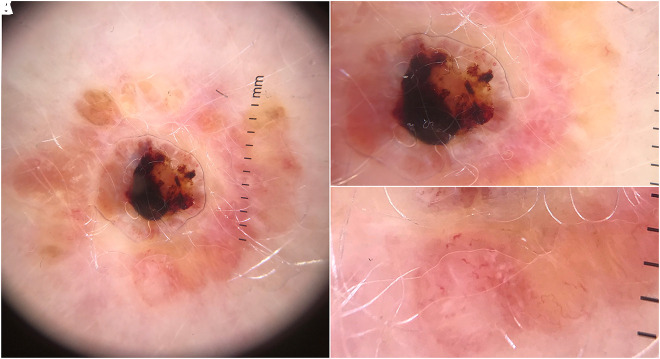
(A) Handheld polarized dermoscopy shows central yellow to orange structureless areas, central hemorrhagic crust, peripheral arrangement of large yellow to orange clods and structureless areas, and (B,C) linear, curved, and complex looped vessels.

**Figure 3:**
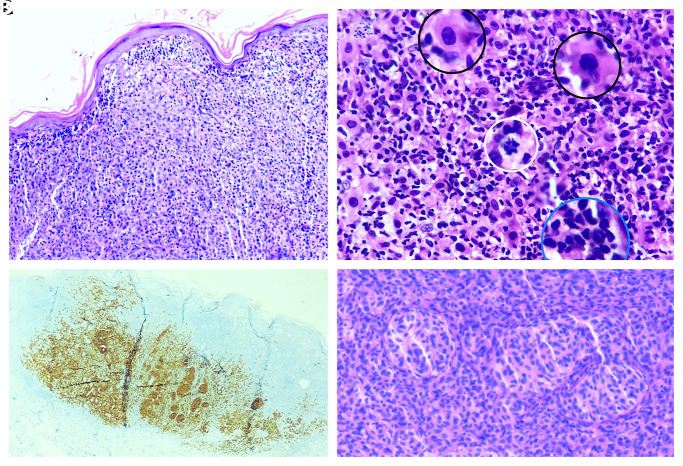
Histopathological examination. (A) Epidermal consumption, superficial dermal mononuclear inflammatory infiltration, and atypical melanocytic infiltration (H&E, ×200). (B) High power shows malignant melanocytes (black circles), atypical mitosis (white circle), and mononuclear inflammatory infiltration (blue circle) (H&E, ×400). (C) Diffuse staining with Melan-A (×200). (D) Bland-looking dermal nests indicating underlying nevus (H&E, ×400).

A 32-year-old female patient with OCA1 presented with a cutaneous lesion that had been enlarging for about a year. She stated that there was an asymptomatic pinkish plaque existing since childhood at the same location. The patient had hypomelanotic skin, blonde hair, blue-gray irides, and bilateral nystagmus. Dermatological examination revealed multiple pinkish papules surrounding a main central tumoral lesion over the right forearm (Figure 1). Dermoscopy of the lesions showed central yellow to orange structureless areas, central hemorrhagic crust, a peripheral arrangement of large yellow to orange clods and structureless areas, and polymorphous vessels including linear, curved, and complex looped vessels (Figure 2). An incisional biopsy was made with preliminary diagnoses of cutaneous sarcoidosis, leishmaniasis, and cutaneous lymphoma. Histopathological examination of the incisional biopsy specimen revealed epidermal consumption, superficial dermal mononuclear inflammatory infiltration, a few bland-looking dermal nevus nests, and atypical melanocytic infiltration filling the lower half of the papillary dermis and reticular dermis with numerous mitoses, including atypical ones. No maturation was observed. Breslow thickness was 2.3 mm. Immunohistochemically, tumor cells were stained with HMB-45, Melan-A, and S-100 (Figure 3). A diagnosis of amelanotic nodular melanoma was made and a total excision with 2-cm margins was performed. No lymph node involvement and metastasis were detected.

## Conclusions

Melanomas in patients with OCA are rare and usually amelanotic. Unfamiliar clinical and dermoscopic findings may cause diagnostic delay, which is usually associated with poor prognosis. Furthermore, nevi in patients with OCA may have a similar dermoscopic pattern to that described for amelanotic melanoma [1].

Only a few studies have reported dermoscopic findings of melanoma in patients with OCA. Irregular dots, globules, blue-white veil, peripheral arciform vessels, and milky red areas were the dermoscopic features reported in the study of Caldarola et al [2]. The present case had a different dermoscopic presentation. A central core of orange structureless areas surrounded by large yellow to orange clods and polymorphous vessels including linear, curved, and complex looped ones composed the main picture. All previously described cases of amelanotic melanomas in patients with OCA predominantly demonstrated a polymorphous vessel pattern.

Patients with OCA may have numerous pinkish lesions, and it can be very difficult to differentiate melanoma from benign lesions. In this context, dermoscopic examination can be life-saving. Dermoscopic analysis in patients with OCA is mainly based on the vascular structures because of the lack of pigmentation [1]. Pink nevi usually demonstrate only curved and comma vessels, while isolated lesions with dotted and linear vascular structures should prompt to exclude melanoma and other malignancies. Knowing the possible dermoscopic presentations of melanoma and the other tumors in patients with OCA may lead to early diagnosis and favorable prognostic outcomes. Age, location, ulceration, Breslow thickness, mitosis rate, and vascular invasion are the indicators of prognosis of melanoma in OCA as they are in any other type of melanoma.
